# 1725. Differences in serotype 3 antibody response and antibody functionality compared to serotype 19A following 13-valent pneumococcal conjugate immunization in children

**DOI:** 10.1093/ofid/ofad500.1557

**Published:** 2023-11-27

**Authors:** Naoko Fuji, Minh Pham, Ravinder Kaur, Michael Pichichero

**Affiliations:** Rochester General Hospital Research Institute, Rochester, New York; San Francisco State University, San Francisco, California; Rochester General Hospital Research Institute, Rochester, New York; Rochester General Hospital Research Institute, Rochester, New York

## Abstract

**Background:**

Background. 13-valent pneumococcal conjugate vaccine (PCV13) includes serotype 3 and 19A. However, prevention of infections with PCV13 may be less effective against serotype 3 than 19A. In young children, determine differences in IgG antibody levels, functionality of antibody by OPA and antibody avidity for serotype 3 compared to 19A following PCV13 immunization; determine IgG antibody levels following natural immunization for serotype 3 and 19A; reassess effectiveness of PCV13 against serotype 3 and 19A in prevention of acute otitis media (AOM) and colonization.

**Methods:**

Samples were secured during a prospective longitudinal study conducted in Rochester NY of children age 6-36 months old. Pneumococcal detection was determined by culture. 713 serum samples were tested for antibody levels by ELISA, 68 serum for functional antibody by OPA, and 47 serum for antibody avidity by thiocyanate bond disruption. Age effects on antibody levels was modeled by generalized estimating equations. PCV13 effectiveness was assessed compared to pre-PCV13.

**Results:**

The proportion children who did not reach protective threshold against IPD (< 0.35 µg/ml) after PCV13 immunization was higher for serotype 3 antibody compared to 19A. PCV13-induced antibody levels to serotype 3 were1.4 to 3.4 fold lower than 19A. OPA titers were not significantly different for serotype 3 compared to 19A, but antibody avidity at child age 6 and 15 months was significantly lower. Serotype 3 natural- immunized children showed a positive trend of increase in antibody level as children got older. In contrast, PCV13-immunized children showed a negative trend of antibody levels with age. For serotype 19A, both natural immunization and PCV13 immunization showed a positive trend in antibody levels over age. PCV13 effectiveness was not identified in preventing AOM or colonization for serotype 3 but effectiveness of 19A was re-confirmed.

**Conclusion:**

Conclusions: PCV13 elicits lower antibody levels and lower avidity antibody to serotype 3 than 19A. PCV13 does not elicit antibody levels that increase over age despite a booster dose for serotype 3, as occurs for serotype 19A. Post PCV13-induced antibody levels for serotype 3 may be insufficient to prevent AOM and colonization in young children.

**Disclosures:**

**All Authors**: No reported disclosures

